# The supplemental value of mammographic screening over breast MRI alone in BRCA2 mutation carriers

**DOI:** 10.1007/s10549-020-05642-1

**Published:** 2020-04-24

**Authors:** Inge-Marie Obdeijn, Ritse M. Mann, Claudette C. E. Loo, Marc Lobbes, Eleonora M. C. Voormolen, Carolien H. M. van Deurzen, Geertruida de Bock, Hereditary Breast Ovarian Cancer Research Group Netherlands (HEBON), Maartje J. Hooning

**Affiliations:** 1 Department of Radiology and Nuclear Medicine, Erasmus University Medical Center, Rotterdam, The Netherlands; 2 Department of Radiology and Nuclear Medicine, Radboud University Medical Center, Nijmegen, The Netherlands; 3 Department of Radiology, The Netherlands Cancer Institute, Amsterdam, The Netherlands; 4 Department of Medical Imaging, Zuyderland Medical Center, Sittard-Geleen, The Netherlands; 5 Department of Radiology and Nuclear Medicine, University Medical Center, Maastricht, The Netherlands; 6 GROW School for Oncology and Developmental Biology, Maastricht University, Maastricht, The Netherlands; 7 Department of Radiology and Nuclear Medicine, Leiden University Medical Center, Leiden, The Netherlands; 8 Department of Pathology, Erasmus University Medical Center, Rotterdam, The Netherlands; 9 Department of Epidemiology, University Medical Center, Groningen, The Netherlands; 10 Department of Medical Oncology, Erasmus MC Cancer Institute, Rotterdam, The Netherlands

**Keywords:** BRCA mutation carriers, Screening, MRI, Mammography

## Abstract

**Purpose:**

BRCA2 mutation carriers are offered annual breast screening with MRI and mammography. The aim of this study was to investigate the supplemental value of mammographic screening over MRI screening alone.

**Methods:**

In this multicenter study, proven BRCA2 mutation carriers, who developed breast cancer during screening using both digital mammography and state-of-art breast MRI, were identified. Clinical data were reviewed to classify cases in screen-detected and interval cancers. Imaging was reviewed to assess the diagnostic value of mammography and MRI, using the Breast Imaging and Data System (BI-RADS) classification allocated at the time of diagnosis.

**Results:**

From January 2003 till March 2019, 62 invasive breast cancers and 23 ductal carcinomas in situ were diagnosed in 83 BRCA2 mutation carriers under surveillance. Overall screening sensitivity was 95.2% (81/85). Four interval cancers occurred (4.7% (4/85)). MRI detected 73 of 85 breast cancers (sensitivity 85.8%) and 42 mammography (sensitivity 49.9%) (*p* < 0.001).

Eight mammography-only lesions occurred. In 1 of 17 women younger than 40 years, a 6-mm grade 3 DCIS, retrospectively visible on MRI, was detected with mammography only in a 38-year-old woman. The other 7 mammography-only breast cancers were diagnosed in women aged 50 years and older, increasing sensitivity in this subgroup from 79.5% (35/44) to 95.5% (42/44) (*p* ≤ 0.001).

**Conclusions:**

In BRCA2 mutation carriers younger than 40 years, the benefit of mammographic screening over MRI was very small. In carriers of 50 years and older, mammographic screening contributed significantly. Hence, we propose to postpone mammographic screening in BRCA2 mutation carriers to at least age 40.

## Introduction

Women with a BRCA1 or BRCA2 mutation have a strongly elevated risk of developing breast cancer [1]. Therefore, annual screening with MRI and mammography starting at young age is advised for women who do not opt for bilateral prophylactic mastectomy, though the optimal screening regimen is not set yet.

In women with a high familial or genetic risk, breast MRI was originally introduced as an adjunct to mammographic screening. However, increasing breast MR expertise and technologic advances over the years have made that breast MR nowadays outperforms mammography in the early detection of invasive breast cancer as well as in the detection of ductal carcinoma in situ (DCIS) [2]. Recent studies demonstrate that, especially in BRCA1 mutation carriers under the age of 40, there is little benefit of mammographic screening when MRI screening is also performed [3–5]. Furthermore, the exposure to low-dose ionizing radiation as from annual mammographic screening might be more harmful in BRCA mutation carriers. The BRCA gene is involved in the pathway of repair of DNA double strand breaks. In BRCA gene mutation carriers, the impaired function of this pathway may lead to a higher risk of radiation-induced breast cancer [6–8].

As a consequence, in 2018, the Dutch screening guidelines were modified for BRCA1 mutation carriers, nowadays starting with supplemental biennial mammography only from the age of 40 [9].

From the point of breast cancer risk and radiation sensitivity, it would be reasonable to screen BRCA1 and BRCA2 mutation carriers according to the same protocol. However, characteristics of cancers detected in BRCA2 carriers differ significantly from those detected in BRCA1 carriers. While BRCA1 carriers predominantly present with high-grade hormone receptor-negative invasive breast cancer, BRCA2 mutation carriers have more luminal breast cancers and a higher proportion of DCIS, sometimes only detected as mammographic calcifications [10–12]. Consequently, in BRCA2 carriers, supplemental annual mammography from the age of 30 is still regarded as the standard of care.

The aim of this study was to assess whether this bi-modal screening protocol (i.e., with digital mammography from age 30 and state-of-art MRI) is still appropriate in BRCA2 mutation carriers. For this, we evaluated the mode of detection in BRCA2 mutation carriers who developed breast cancer while under surveillance.

## Patients and methods

### Patients

In this retrospective study, we included women with a proven BRCA2 mutation who developed breast cancer during surveillance with digital mammography and breast MRI in one of the five participating Dutch university hospitals (Erasmus University Medical Center Rotterdam, Radboud University Medical Center Nijmegen, Maastricht University Medical Center, University Medical Center Leiden, and University Medical Center Groningen) or in the Netherlands Cancer Institute in Amsterdam. We included consecutive series of BRCA2 breast cancer cases provided by the databases of the family breast cancer clinics of Erasmus University Medical Center Rotterdam and Radboud University Medical Center Nijmegen. For the other centers, we included BRCA2 breast cancer cases provided by the collaborative group on Hereditary Breast and Ovarian Cancer in The Netherlands (HEBON) [13].

Inclusion started after introduction of digital mammography which was between January 2003 (Radboud University Medical Center Nijmegen) and September 2006 (Erasmus University Medical Center Rotterdam). The inclusion period ended in March 30, 2019.

All BRCA2 breast cancer patients provided written informed consent.

Women in whom a BRCA2 mutation was determined after breast cancer diagnosis and BRCA2 mutation carriers with breast cancer detected in specimen from prophylactic mastectomy were excluded from the analysis. We also excluded women in whom only one of the screening methods was used.

Women with a prior history of breast cancer were included.

### Methods

#### Age

We divided the women in two age groups: women diagnosed with breast cancer before age 40 and women diagnosed with breast cancer at age 40 and older. We chose this age limit for two reasons. Firstly, previous research in women at increased risk showed a very low added value of mammography in the screening of women under 40 years of age, albeit series were too small to provide specific information for women with BRCA2 mutations. Secondly, the breast cancer risk from low-dose ionizing radiation as from annual mammographic screening is strongly dependent on age at exposure with higher risks at younger ages [5, 14, 15].

#### Breast MRI

According to the Dutch guidelines [9], women with a proven BRCA2 mutation were offered annual breast MRI screening from age 25 till age 60 and annual mammographic screening starting at age 30. After age 60, breast cancer screening is continued with annual mammography, or, in case of dense breast tissue, with annual screening alternating mammography and MRI.

Breast MRI was performed in each center using local protocols that all met the requirements of currently accepted international guidelines [16], including at least three high-resolution T1-weighted acquisitions obtained before, early (90s), and late after (5–7 min) contrast administration. Dedicated analysis software (allowing for the creation of maximum intensity projections, signal intensity versus time curves, and color-coded overlays of enhancement patterns) and MR-guided biopsy tools were available in all centers. All centers had extensive experience with screening of women with familial or genetic predisposition for breast cancer.

#### Data collection

The images and related reports of all patients at the time of diagnosis were reviewed by one or two of the dedicated breast radiologists (IMO, RM, CL, ML, NV) in order to assess whether the breast cancers were screen-detected or interval cancers and whether the cancers were visible on mammography and MRI. One radiologist (IMO) reviewed nearly all cases. The mammograms and breast MRI examinations were reported at the time of diagnosis according to the Breast Imaging Reporting and Data System (BI-RADS) [17]. The BI-RADS classifications (both for mammography and MRI) allocated at the time of diagnosis were used for this evaluation. BI-RADS classifications 0, 3, 4, and 5 were defined as positive findings because additional work-up was indicated.

A screen-detected breast cancer was defined as a breast cancer found during a screening round by breast MRI and/or mammography. If a breast cancer was identified clinically (became palpable or caused other symptoms) in between two screening rounds, it was considered an interval cancer.

The pathology reports were evaluated for tumor characteristics. In women who underwent primary surgical treatment, the largest diameter of the tumor as mentioned in the pathology report was recorded to indicate size. In case of multifocal or multicentric disease, the size of the largest cancer was registered. In patients treated with neo-adjuvant chemotherapy, the largest diameter measured on the pre-therapeutic MRI was recorded as the size of the breast cancer.

### Statistical analysis

Sensitivity for both screening modalities (breast MRI and mammography) was assessed separately. For the comparison of the screening modalities, we used the data of patients for whom results were available for both modalities at the time of diagnosis. The differences in sensitivity between the two were tested by a McNemar’s test for paired proportions.

We also compared sensitivity for breast MRI and mammography separately for the two age groups defined above (women diagnosed before age 40 and women diagnosed at age 40 years and older). The differences in sensitivity between age groups were tested using Fisher’s exact test.

A two-sided *p* value of lower than 0.05 was regarded as statistically significant. Statistical analyses were performed using STATA (STATA version 15.1).

## Results

From January 2003 to March 2019, 83 BRCA2 mutation carriers were diagnosed with 85 breast cancers while under surveillance with both MRI and digital mammography. In the majority of cases (in 75 of 85 cases), MRI and mammography were performed simultaneously or within a short period of time. One woman presented with a synchronous contralateral breast cancer, another woman had a metachronous contralateral breast cancer. Mean age at diagnosis was 49.3 years (range 27–70); 17 breast cancers were diagnosed before age 40 (20.0%).

Sixty-two invasive breast cancers with a mean size of 10.1 mm (range 3–27 mm) and 23 DCIS lesions were detected. Tumor characteristics can be found in Table 1. The majority, 68.2% (58/85) of breast cancers, was detected in an early stage (DCIS or invasive breast cancer ≤ 10 mm). Lymph node status was determined to be positive in 12 of 61 women in whom at least a sentinel lymph node biopsy was performed (19.7%).

**Table 1:** Tumor characteristics of 85 breast cancers

Histologic type	Number
DCIS *n* = 23 (27.1%)	Histological grade DCIS
	Grade 1 0
	Grade 2 12
	Grade 3 11
Invasive breast cancer *n* = 62 (72.9%) (Invasive breast cancer with DCIS component *n* = 25)	Histology-invasive breast cancer Invasive breast cancer no special type 54 Invasive lobular carcinoma 6 Other 2
	Histological grade invasive breast cancer Bloom and Richardson Grade 1 7 Bloom and Richardson Grade 2 30 Bloom and Richardson Grade 3 25
	Size invasive breast cancer ≤ 10 mm 35 (56.5%) > 10 ≤ 20 mm 25 (40.3%) > 20 mm 2 (3.2%)
	Receptor status invasive breast cancer *n* = 59* ER+ and/or PR+, HER2− 42 (71.2%) ER+, PR+, HER2+ 3 (5.1%) ER−, PR−, HER2+ 3 (5.1%) ER+, PR_, HER2+ 1 (1.7%) Triple− 10 (16.9%)
Nodal status *n* = 61***	N+ 19.7%** (12/61) N− 80.3% (49/61)

*DCIS* ductal carcinoma in situ, *HER2* human epidermal growth factor receptor 2

*Her2 status not available in 3 patients

**Including 4 cases with micrometastases

*** Nodal status unknown in one patient with invasive breast cancer

Overall, 81 out of 85 breast cancers were screen-detected, resulting in an overall screening sensitivity of 95.2% (81/85). Four interval cancers occurred (4.7% (4/85)) in women aged 39, 42, 50, and 51 years with sizes of, respectively, 15, 12, 17, and 19 mm. Based upon the re-evaluation of the imaging examinations, two of them were retrospectively visible on MRI. Three of 4 interval cancers were grade 3 triple-negative breast cancer.

MRI detected 73 of 85 breast cancers (sensitivity 85.8%) and mammography 42 (sensitivity 49.9%) (*p* value < 0.001). For invasive breast cancer, MRI performed significantly better than mammography (sensitivity, respectively, 90.3% (56/62) versus 43.5% (27/62), (*p* < 0.001)). For the detection of DCIS, the difference was not statistically significant (sensitivity, respectively, 73.9% (17/23) and 65.2% (15/23) (*p* = 0.791)).

The screening sensitivity of the combination of imaging modalities, and the screening sensitivity of breast MRI and mammography separately, was also determined for the pre-defined age groups (Table 2).

**Table 2:** Screening sensitivity according to screening modality and age at diagnosis

Sensitivity	All breast cancers* n* = 85	Breast cancers diagnosed before age 40 *N* = 17 (20.0%)	Breast cancers diagnosed at age ≥ 40 years *n* = 68 (80.0%)	*p*
**Combined screening**				
All breast cancers	95.2% (81/85)	94.1% (16/17)	95.6% (65/68)	1.000
DCIS	100% (23/23)	100% (6/6)	100% (17/17)	1.000
Invasive breast cancer	93.5% (58/62)	90.1% (10/11)	94.1% (48/51)	1.000
**MR screening**				
All breast cancers	85.8% (73/85)	88.2% (15/17)	85.3% (58/68)	1.000
DCIS	73.9% (17/23)	83.3% (5/6)	70.6% (12/17)	1.000
Invasive breast cancer	90.3% (56/62)	90,9% (10/11)	90.1% (46/51)	1.000
**X screening**				
All breast cancers	49,4% (42/85)	47.1% (8/17)	50.0% (34/68)	1.000
DCIS	65.2% (15/23)	50.0% (3/6)	70.6% (12/17)	0.621
Invasive breast cancer	43.5% (27/62)	45.4% (5/11)	43.1% (22/51)	1.000

Thirty-nine breast cancers were detected by MRI only (45.8% (39/85)) and 8 by mammography only (9.4% (8/85) (Table 3)). It should be noted that only two of the cancers detected with mammography only were invasive. One of the mammography-only breast cancers was diagnosed before age 40. It concerned a 6 mm DCIS grade 3 in a 38-year-old woman, retrospectively also visible on MRI.

**Table 3:** Mammography-only detected breast cancers

No	Age at diagnosis	Tumor type	Grade		Size	Nodal status
1	57	Invasive breast cancer NST with EIC * ER-, PR-, Her2-	2		5 mm	*N*-
2	54	DCIS	3		10 mm	np
3	54	Invasive breast cancer NST with EIC ER+, PR+, Her2−	3		3 mm	*N*-
4	57	DCIS	2		6 mm	np
5	51	DCIS	2		20 mm	np
6	38	DCIS*	3		6 mm	np
7	57	DCIS	3		15 mm	np
8	59	DCIS*	2		14 mm	np

*NST* no special type, *EIC* extensive intraductal component, *np* sentinel node or axillary lymph node dissection not performed

*In retrospect visible on MRI

**Fig. 1:**
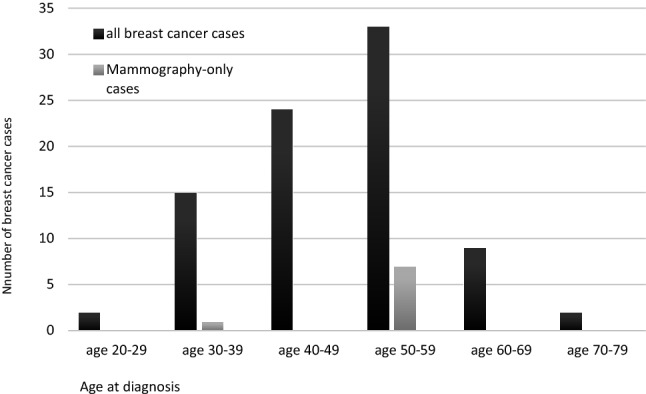
All breast cancer cases and mammography-only breast cancer cases per 10-year age group

The other 7 mammography-only breast cancers were diagnosed in women aged 50 years and older (Table 3 and Fig. 1). This subgroup consisted of 42 women with 44 breast cancers of which 35 were correctly depicted by MRI (sensitivity 79.5% (35/42)). Decombination of MRI and mammography increased the screening sensitivity in this subgroup to 95.5% (42/44).

No mammography-only breast cancers occurred in the subgroup of 24 women diagnosed with breast cancer between age 40 and 49 (Fig. 1).

## Discussion

In this multicenter study, the screening results in BRCA2 mutation carriers showed a high screening sensitivity of 95.2% as well as a high percentage of early-stage breast cancer (68.2%) and a very low fraction of interval cancers (4.7%). As expected, the sensitivity of MRI (85.8%) was significantly higher than the sensitivity of mammographic screening (49.9%) (*p* value < 0.001). The most important finding of the current study is that in the 17 BRCA2 mutation carriers diagnosed with breast cancer before age 40, there was just one mammography-only lesion concerning a 6 mm DCIS grade 3 in a 38-year-old woman (5,9% (1/17)). This lesion was retrospectively visible on MRI and therefore not truly MRI occult. The additional value of mammography is negligibly small in BRCA2 carriers younger than 40 years old.

However, in BRCA2 mutation carriers, aged 50 and older mammographic screening did demonstrate a screening benefit in addition to MR screening. Of the 44 cancers diagnosed above age 50, 7 cancers, concerning predominantly DCIS but also small invasive lesions with an extensive intraductal component, were detected by mammography only, increasing sensitivity in this subgroup from 79.5% (35/44) to 95.5% (42/44) (*p* = 0.0082), indicating that mammography may have supplemental value in this age group.

Strikingly, mammographic screening did not contribute over MRI screening in women diagnose between age 40 and 49 years. The very limited additional value of mammographic screening combined with the increased radiation risks in young mutation carriers [5, 6] and the additional burden placed upon patients by supplemental mammography are arguments to postpone the start of mammographic screening to at least age 40. The lack of screening benefit of mammography over MRI in women between 40 and 49 years of age suggests that mammographic screening might even be postponed to age 50. A prerequisite is that annual MRI screening is performed in a dedicated center.

One could argue that the DCIS cases depicted by mammography-only represent overdiagnosis. However, an argument against overdiagnosis is that all DCIS lesions detected with mammography-only as well as with MRI were grade 2 or grade 3, which seems relevant in this high-risk population. Moreover, it is known that pre-invasive lesions, such as DCIS, are common in BRCA-associated breast cancers [18] and that in high-risk women with DCIS, the prevalence of a BRCA1/2 mutation is high [19]. Yang and colleagues [20] found, in BRCA-associated breast cancers with an invasive and an in situ component, a high concordance of DCIS and invasive phenotypes. These findings may suggest that DCIS, like in sporadic breast cancer, may be considered as a step in the pre-invasive progression pathways in BRCA mutation-related breast cancers.

Phi et al. [3] conducted a meta-analysis of six high-risk prospective screening studies and reported on 72 BRCA2 breast cancer cases. In contrast to our results, one-third (6/18) of breast cancers diagnosed before age 40 years were detected with mammography only. However, in this meta-analysis, the MRI sensitivity was just 50% (9/18) for the younger age group and 73.6% (53/72) for all ages. The poor MRI screening results of this meta-analysis can likely be explained by the fact that some of the included studies were multicenter studies starting with breast MRI screening at the beginning of the MRI-era and, therefore, were conducted in centers with little MRI screening experience. MR screening performance has clearly improved since then [2, 4, 21, 22].

The prospective cohort study of the High Risk Ontario Breast Screening Program [23] included, from July 2011 to December 2016, 8782 high-risk women, of which 1885 were BRCA1/2 mutation carriers. In mutation carriers, younger than 40 years of age the sensitivity of MRI alone was comparable to the combination of MRI and mammography (96.8% vs 100%, *p* 0.99). In carriers aged 50–69 years, combining MRI and mammography increased sensitivity compared with MRI alone (92.7% vs 83.5%, *p* 0.02), which seems in line with our findings. However, outcomes were not given for BRCA1 and BRCA2 mutation carriers separately.

Other studies [12, 24–30] presenting screening results in proven BRCA2 mutation carriers had small numbers, varying between 2 and 25 breast cancer cases. The mammographic benefit in these studies was limited, especially in women diagnosed with breast cancer before age 40. However, the small numbers make it difficult to draw solid conclusions.

Krammer [31] presented the results of 496 BRCA breast cancer cases diagnosed between 1999 and 2013. None of the 211 BRCA2 breast cancers were identified by mammography only. However, at the time of breast cancer diagnosis, nearly half of the participants had clinical symptoms. Therefore, the outcomes are difficult to compare with ours and that of the above-mentioned studies, but underline the observation that MRI seems to detect most of BRCA2-associated breast cancers.

It should be noted that 2 of 4 interval cancers and 3 of 8 mammography-only cancers were in retrospect visible at the MRI scan, though not recalled. While unfortunate, this finding is not unexpected, and in line with previous studies that retrospectively evaluated MRI scans of patients with breast cancer initially reported to be negative [32–34]. The missed lesions and the higher number of mammography-only lesions in this patient population explain the relatively low MRI sensitivity.

The main limitation of our study is its retrospective design. However, to avoid information bias, the original allocated BI-RADS classifications were used. Moreover, we included data from consecutive patient series when possible. Furthermore, although we present a large cohort of BRCA2 breast cancer cases, the sample size is still small. In addition, no information can be provided about the specificity of the screening modalities as we evaluated only breast cancers cases and not a complete screened cohort. However, Vreemann et al. reported a specificity of 84.8% for first-round MRI examinations in BRCA2 carriers, which steeply increases to 97.4% in follow-up rounds, but is still somewhat lower than for mammography (94.4% and 98.8%, respectively) [35]. Likewise Bick et al. reported, for BRCA2 mutation carriers, a specificity of 85.1% in first-round examinations and 92.9% in follow-up examinations using multimodality screening [36]. Further large prospective screening cohorts studies, like the High Risk Ontario Breast Screening Program, are necessary to define the optimal screening protocol for BRCA1 and BRCA2 mutation carriers.

## Conclusions

Mammographic screening appears to have minimal benefit over MRI screening in BRCA2 mutation carriers younger than 40 years and may not overcome the disadvantages of increased radiation risks.

In BRCA2 mutation carriers of 50 years and older, mammographic screening contributed significantly in the detection of early-stage breast cancer. We propose to omit mammographic screening in young BRCA2 mutation carriers and suggest to postpone mammographic screening in BRCA2 mutation carriers to at least age 40, or even to age 50. While the mammography-only lesions were predominantly DCIS one could also consider to perform mammographic screening every two years, starting at age 40.

## Abbreviations

BI-RADS: Breast Imaging Reporting and Data System
DCIS: Ductal carcinoma in situ
HER2: Human epidermal growth factor receptor 2
ILC: Invasive lobular carcinoma
Invasive carcinoma NST: Invasive carcinoma no special type
HEBON: Hereditary Breast and Ovarian Cancer Research Group Netherlands
MRI: Magnetic Resonance Imaging
